# Efficient and reliable establishment of lymphoblastoid cell lines by Epstein-Barr virus transformation from a limited amount of peripheral blood

**DOI:** 10.1038/srep43833

**Published:** 2017-03-08

**Authors:** Natsue Omi, Yuichi Tokuda, Yoko Ikeda, Morio Ueno, Kazuhiko Mori, Chie Sotozono, Shigeru Kinoshita, Masakazu Nakano, Kei Tashiro

**Affiliations:** 1 Department of Genomic Medical Sciences, Kyoto Prefectural University of Medicine, Kyoto, Japan; 2 Department of Ophthalmology, Kyoto Prefectural University of Medicine, Kyoto, Japan; 3 Department of Frontier Medical Science and Technology for Ophthalmology, Kyoto Prefectural University of Medicine, Kyoto, Japan

## Abstract

Lymphoblastoid cell lines (LCLs) transformed by Epstein-Barr virus (EBV) serve as an unlimited resource of human genomic DNA. The protocol that is widely used to establish LCLs involves peripheral blood mononuclear cell isolation by density gradient centrifugation, however, that method requires as much as 5 ml of peripheral blood. In this study, in order to provide a more simple and efficient method for the generation of LCLs, we developed a new protocol using hemolytic reaction to enrich white blood cells for EBV transformation and found that the hemolytic protocol successfully generated LCLs from a small volume (i.e., 0.1 ml) of peripheral blood. To assess the quality of genomic DNA extracted from LCLs established by the hemolytic protocol (LCL-hemolytic), we performed single nucleotide polymorphism (SNP) microarray genotyping using the GeneChip^®^ 100 K Array Set (Affymetrix, Inc.). The concordances of the SNP genotyping resulting from genomic DNA from LCL-hemolytic (99.92%) were found to be as good as the technical replicate (99.90%), and Kappa statistics results confirmed the reliability. The findings of this study reveal that the hemolytic protocol is a simple and reliable method for the generation of LCLs, even from a small volume of peripheral blood.

The Epstein-Barr virus (EBV) is known to infect and transform human B cells into lymphoblastoid cell lines (LCLs) *in vitro*^1^. LCLs serve as an unlimited resource of human genomic DNA, as the established cell lines apparently maintain the genome intact through generations, regardless of the viral genome persisting intracellularly^2^,^3^. In fact, several non-profit depository facilities currently make a huge contribution to the scientific community by storing a number of LCLs and distributing genomic DNA derived from them to researchers upon request^4^,^5^. Moreover, our research group at Kyoto Prefectural University of Medicine (KPUM), Kyoto, Japan consistently collects thousands of blood samples from patients and healthy volunteers, and we have performed several genome-wide association studies (GWAS) using their genomic DNA^6^,^7^,^8^,^9^. With each sample that we obtain, we are also establishing LCLs to serve as a resource of future studies, such as for a resequencing analysis of the disease-associated regions identified by GWAS. However, the procedure for establishing LCLs is often time-consuming and labor-intensive, especially when numerous samples need to be simultaneously handled, due to the complex steps needed to generate LCLs. In addition, we occasionally encounter situations in which the amount of blood that can be collected from a subject is less than 5 ml, i.e., less than the amount needed to establish LCLs via the use of the conventional method involving density gradient centrifugation. Therefore, the aim of this present study was to develop a more simple and effective method for generating LCLs.

At present, the most widely accepted method for the establishment of LCLs utilizes the protocol of density gradient centrifugation in order to prepare peripheral blood mononuclear cells (PBMCs) from peripheral blood (hereafter referred to as the “gradient protocol”) before EBV infection^10^,^11^,^12^,^13^. For the gradient protocol, there are a wide variety of commercially available reagents that allow researchers to separate PBMCs from the different layers of the other components of blood based on each density^13^,^14^,^15^. The gradient protocol typically requires 5 ml or more of peripheral blood in order for it to be overlaid onto the gradient-making reagent^16^,^17^,^18^. Moreover, post centrifugation, the method required to collect the interface layer, which contains PBMCs, is complex. An alternative method to establish LCLs has been reported, which involves adding EBV-containing culture supernatant on white blood cells (WBCs) consisting of PBMCs and granulocytes, which are prepared by removing the lysed red blood cells from the peripheral blood by hemolytic reaction (hereafter referred to as the “hemolytic protocol”)^19^. However, the initial volume of peripheral blood needed in that method is still 5 ml.

Thus, the aim of this present study was to develop a simple and efficient protocol for the establishment of LCLs, specifically focused on the generation LCLs from a limited amount of peripheral blood. In addition, in order to assess and confirm that the quality of the genomic DNA extracted from LCLs established by this novel method using hemolytic reaction (LCL-hemolytic) is as good as genomic DNA extracted from peripheral blood and genomic DNA extracted from LCLs established by the conventional method using density gradient centrifugation (LCL-gradient), we performed single nucleotide polymorphism (SNP) microarray genotyping using the GeneChip^®^ 100 K Array Set (Affymetrix, Inc., Santa Clara, CA), and then compared the concordance of genotyping results using each of the genomic DNAs.

## Results

### Comparison of cell recovery, viability, and proportion of cell components

In this study, prior to initiating the comparison of cell recovery, cell viability, and the proportion of cell components, a preliminary test was performed to assess the effect of time during which peripheral blood samples were kept before being used as starting materials on the period of days required for successful LCL generation. In addition, the effect of temperature in which peripheral blood samples were kept before being used as starting materials on the period of days required for successful LCL establishment was also assessed (Supplementary Fig. S1). No significant difference was observed between peripheral blood samples stored at either 4 °C or 25 °C. In addition, no difference was observed between peripheral blood samples stored for 3 days and those used on day 0; i.e., the day the peripheral blood was obtained from the volunteer subjects (Supplementary Fig. S1a). After 7-days storage, the period of days required for successful LCL establishment was found to be prolonged (Supplementary Fig. S1b). Since up to 3-days storage produced no detrimental effect, we theorized that the storage time of within day 0 would also produce no detrimental effect. However, all experiments performed in this study were initiated on day 0.

**Figure 1:**
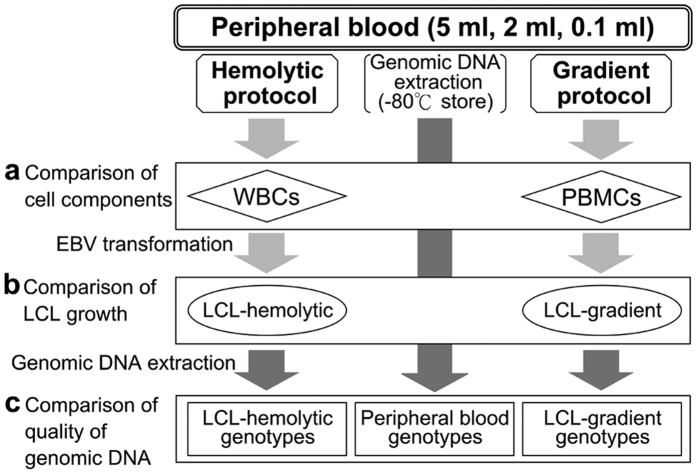
Study design for the comparative experiments. To evaluate the efficiency and reliability of the hemolytic protocol (flowchart, left side), a quantitative comparison was made with the gradient protocol (flowchart, right side) by changing the starting blood volume (5 ml, 2 ml, or 0.1 ml) with respect to the following three categories: (**a**) Comparison of the features of the cells (cell recovery, viability, and proportion of cell components) of the isolated WBCs prepared by the hemolytic protocol or PBMCs prepared by the gradient protocol, (**b**) Comparison of the growth curves of establishing LCLs (ellipse) from WBCs (LCL-hemolytic) or PBMCs (LCL-gradient), and (**c**) Comparison of the quality of genomic DNA (rectangle) based on the SNP genotype data (central arrow).

As shown in Fig. 1a, we first examined the cell properties isolated by hemolytic or gradient protocols using a total of 120 samples from sample groups #1, #2, and #3 (Table 1a). Cell recovery via the gradient protocol was found to be significantly lower than that via the hemolytic protocol (Table 2). No significant difference in cell viability of the isolated cells was found between the two protocols, i.e., in regard to initiating with 5 ml or 2 ml of peripheral blood (*P* > 0.05). When starting with 0.1 ml of peripheral blood, the viability of the isolated cells via the gradient protocol was slightly lower than that via the hemolytic protocol (Table 2; *P* < 0.01). The values of standard deviation (SD) for WBCs seemed to be relatively stable when compared with those of PBMCs, and this was confirmed by assessing the normality of the cell viabilities of WBCs (*P* = 0.769) and PBMCs (*P* < 0.001) by the Shapiro-Wilk test, thus suggesting that the hemolytic protocol is technically stable.

In order to determine which cell types were isolated, each cell fraction was stained and categorized, based on the morphology, as Neutrophils, Eosinophils, Basophils, Lymphocytes, and Monocytes. The major cell component populations isolated by the hemolytic protocol were neutrophils and lymphocytes, irrespective of the different starting volumes of peripheral blood (Table 3). As for the gradient protocol, the major cell component population was lymphocytes. However, the proportion of lymphocyte population decreased to less than 70% when started from 0.1 ml of peripheral blood (Table 3), probably due to the incorporation of neutrophils (~30%) while recovering a thin PBMC layer after density gradient centrifugation.

### Comparison of LCL growth

**Figure 2:**
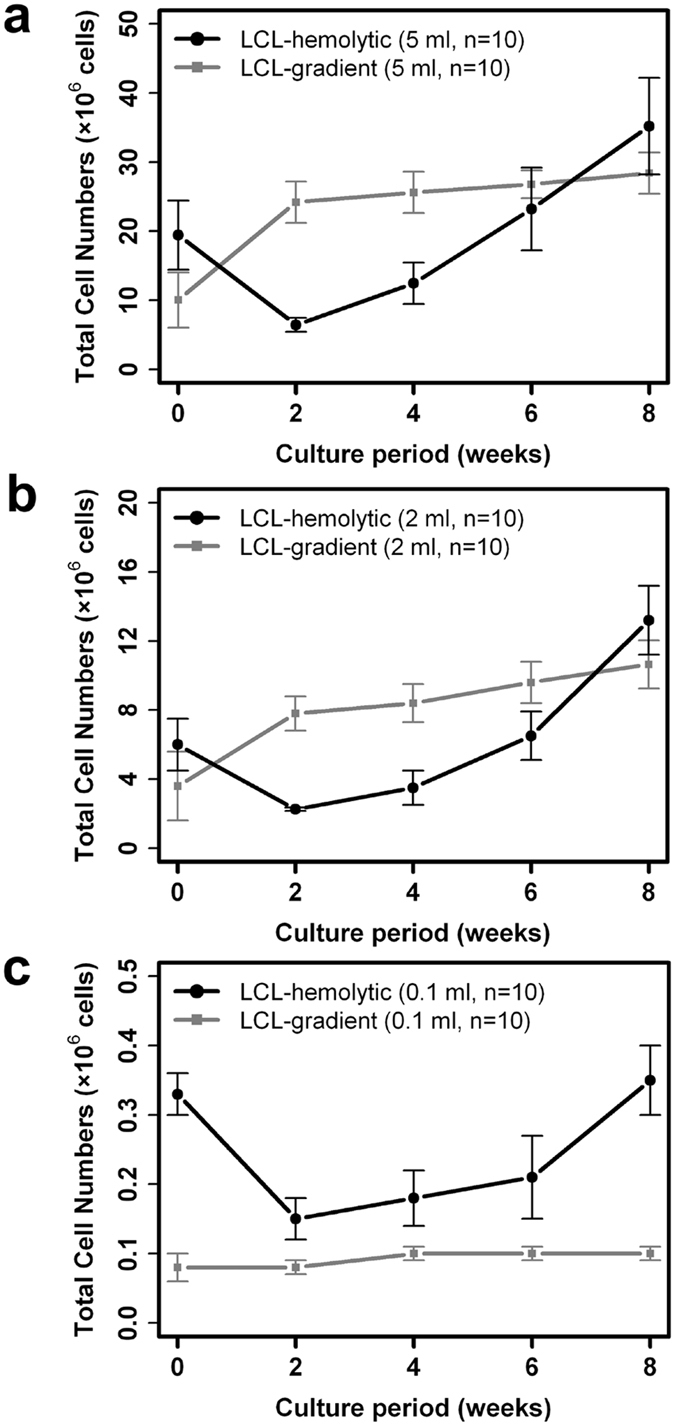
Growth curve of LCLs established from different blood volumes. The LCLs were established by either hemolytic (LCL-hemolytic, solid black circle) or gradient (LCL-gradient, solid gray square) protocol from the peripheral blood of 5 ml (**a**), 2 ml (**b**), or 0.1 ml (**c**). Each LCL was cultured until 8 weeks and observed at 2-week intervals. Each point indicates the average and standard error bar of 10 LCLs. The vertical axis indicates the total number of viable cells and the horizontal axis indicates the weeks post EBV infection.

The growth rate of LCLs established by each protocol (Fig. 1b) was compared. In total, 60 LCLs were generated by either hemolytic (LCL-hemolytic) or gradient (LCL-gradient) protocol using the samples from sample groups #4, #5, and #6 (Table 1b). When the initial volume of peripheral blood was sufficient (≥2 ml), both protocols effectively generated LCLs in 8 weeks (Fig. 2a,b). Cells prepared by each protocol showed unique growth curves. In LCL-gradient, rapid growth was observed during the initial 2 weeks, and a subsequent steady growth of LCLs was observed at close to 8 weeks, as is observed in a general LCL culture (Fig. 2a,b, solid gray square). In LCL-hemolytic, after continuous decrease of total cell number during the initial 2 weeks, rapid recovery of LCL growth was observed through 2 to 8 weeks (Fig. 2a,b, solid black circle). However, when starting with 0.1 ml of peripheral blood, the LCL-gradient failed to expand throughout the observation period, possibly due to the low number of initial viable PBMCs isolated by density gradient centrifugation (Fig. 2c, solid gray square). In contrast, LCL-hemolytic showed the above-described growth curve even when started from a small initial cell number (Fig. 2c, solid black circle), thus suggesting the usefulness of the hemolytic protocol for establishing LCLs from a limited amount of peripheral blood. It is important to note that although LCL-hemolytic initiated from 0.1 ml of peripheral blood required a somewhat longer culture period (i.e., 12 weeks, as opposed to 6 to 8 weeks), it was always possible to obtain a sufficient amount (i.e., a few micrograms) of genomic DNA.

### Comparison of genotypes

In order to evaluate the influence of EBV infection and transformation to the genomic DNA of LCLs, SNP genotype data obtained from genomic DNA derived from peripheral blood and that obtained from genomic DNA derived from LCL-hemolytic were compared (Fig. 1c). For the evaluation, 24 samples were used from sample groups #7, #8, and #9 (Table 1c). The yield and the ratio of A260/280 absorbance of genomic DNAs from the 24 samples was found to be high enough for use in the microarray experiments (Table 4). The periods of each sample stored at −80 °C until isolation of DNA were as follows: #7: 136 weeks (peripheral blood) or 130 weeks (LCL), #8: 60 weeks (peripheral blood) or 52 weeks (LCL), and #9: 10 weeks (peripheral blood). As a result of quality control for SNP genotype data (see Supplementary Note and Supplementary Fig. S2), the concordance rate of technical replicates (Supplementary Fig. S3A) was 99.90% (Table 5), thus indicating that an error rate of approximately 0.1% spontaneously occurred during the 100K-microarray genotype experiments.

Given the above background technical replication data, the concordance rates between the data from genomic DNA from LCLs and that from peripheral blood DNA (Supplementary Fig. S3B) were extremely high (approximately 99.90%) in both the hemolytic and gradient protocols (Table 5). Moreover, when using the hemolytic protocol, the concordance rate remained constant when the starting volume of peripheral blood was reduced to 2 ml (99.90%), and remained high enough even when reduced down to 0.1 ml (99.82%) (Supplementary Fig. S3C and Supplementary Table S1). These results were also confirmed by the Kappa statistics (also known as “Cohen’s kappa coefficient”) (Table 5 and Supplementary Table S1).

**Figure 3:**
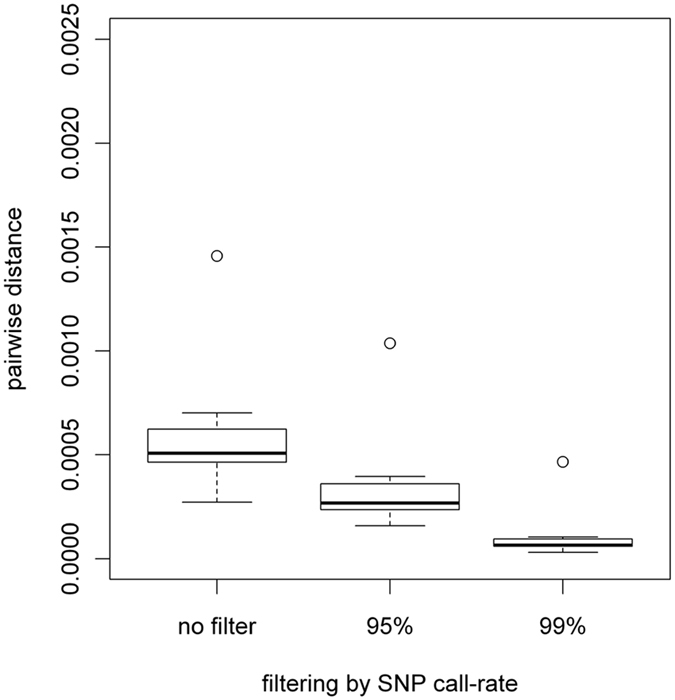
Genotype concordance by pairwise analysis. Genotype concordance between the genomic DNA from LCL-hemolytic and the peripheral blood, from which the LCLs were established, is shown as box plots with median values and interquartile range of pairwise distances. These box plots were generated using 9 samples from sample groups #7, #8, and #9 (Table 1). When the LCL-hemolytic samples maintain the genomic DNA intact, the pairwise distance should be close to 0. The vertical and horizontal axes indicate pairwise distance and the filtering conditions, respectively.

Moreover, the genotype concordance was also analyzed by measuring a pairwise distance between the data derived from the LCL-hemolytic and its peripheral blood (Fig. 3). As a result, the distance obviously became closer to zero when the stringency of the SNP filter (call-rate per SNP) was increased up to >99%.

Taken together, these results suggest that the genomic DNA derived from LCLs established by the hemolytic protocol had a minimum effect of EBV transformation and was sustainable for practical use.

## Discussion

The findings in this present study demonstrate that the hemolytic protocol is a reliable method for establishing LCLs, even when the initial amount of peripheral blood is limited, thus providing an advantage in terms of use for pediatric research and for patients in whom a sufficient amount of peripheral blood is difficult to obtain. Moreover, this method provides an advantage over the conventional gradient protocol, as it allows for an increased number of samples to be enrolled for genetic analysis.

It is important to note that when using the hemolytic protocol, the peripheral blood needs to be treated with a chilled hypotonic buffer for 15 minutes on ice in order to burst erythrocytes. As shown in Table 2, the viability of WBCs isolated by the hemolytic protocol was slightly lower than that of PBMCs isolated by the gradient protocol, although the difference, if any, was small enough not to influence on the following further analysis. In fact, the results showed that it was possible to establish LCLs efficiently via the hemolytic protocol (Fig. 2). In order to evaluate the influence of EBV infection and transformation to the genomic DNA of LCLs, the SNP genotype data obtained from genomic DNA derived from peripheral blood and that obtained from genomic DNA derived from LCL-hemolytic were compared (Table 5, Supplementary Table S1, Supplementary Note). Our findings showed that the concordance rate of the genotype data between the genomic DNA derived from LCLs and its peripheral blood turned out to be sufficiently high (>99.80%) in light of the concordance rate of the technical replicates (99.90%) (Table 5). Moreover, the obtained genotype data of LCLs appeared to be useful for practical use by applying appropriate QC filters (Fig. 3), which was comparable to the findings of the previous study^12^, thus indicating that the hemolytic protocol is a rapid and practical method for establishing LCLs by EBV transformation.

It is also important to note that when using the hemolytic protocol, the PBMCs and granulocytes need to be co-cultivated during EBV transformation. Although that can be a concern, the findings of this study clearly shown that the hemolytic protocol is superior to the gradient protocol for establishing LCLs from 0.1 ml of peripheral blood. In this study, we found that the viability was significantly higher for WBCs than for PBMCs (Table 2), thus suggesting the effectiveness of the hemolytic protocol as a cell purification method for the generation of LCLs. When we examined the cell components of each fraction (Table 3), the content of PBMCs isolated by the gradient protocol was found to be greatly changed when the initial volume of peripheral blood was reduced down to 0.1 ml; i.e., the proportion of lymphocyte population decreased from 90% to less than 70% while the neutrophils increased to ~30%, probably due to the handling while collecting the PBMC layer. Putting the low recovery rate of the gradient protocol (~15%) together (Table 2), the inclusion of neutrophils may result in an insufficient number of B lymphocytes for EBV infection, and thus fail to expand as an LCL (Fig. 2c, solid-gray square). In contrast, since the procedure of the hemolytic protocol simply adopts the removal of erythrocytes, the cell density, as well as the content of enriched PBMCs, remained consistent without being affected by the initial volume of peripheral blood (Tables 2 and 3). Therefore, we feel that this simple and efficient procedure provides an advantage over the gradient protocol.

After infecting EBV to each isolated cell fraction, we observed distinct growth curves of LCLs based on the different protocols (Fig. 2). The LCLs established by the gradient protocol showed rapid growth for the first 2 weeks, as long as the initial cell number was sufficient (Fig. 2a,b), whereas the cell number drastically reduced in the first 2 weeks for LCLs established by the hemolytic protocol in all conditions (Fig. 2a–c). Since the initial pool of WBC fraction consisted of ~43% of lymphocytes together with ~52% of neutrophils (Table 3), this reduction appeared to be reflecting the cell number of more than 50% of the cell population that were not susceptible to EBV and eventually died within 2 weeks. On the other hand, the LCLs seemed to be consistently viable, which was apparent from the conversion into the growth phase of the LCLs after 2 weeks. Since the total cell number was approximately 0.3 × 10^6^ cells at the time of EBV infection when starting with 0.1 ml of peripheral blood (Fig. 2c, solid-black circle), it indicated that the total number of lymphocytes should be less than 0.15 × 10^6^ cells at that time, although the number was still approximately twice as many as the number of lymphocytes prepared by the gradient protocol. This finding suggests that the limit of the total cell number for an efficient LCL generation was around 1 × 10^5^ cells under our experimental conditions.

Although we are generally able to obtain a sufficient amount of blood necessary for any downstream experiments from each patient or volunteer, we sometimes encounter subjects in whom it is difficult to obtain a sufficient amount of peripheral blood. For example, in approximately 1% of the total number of volunteers in our cohort, including not only elderly but also young subjects, we had difficulty obtaining the biological samples needed for our experiments. In order to make good use of those samples, we therefore attempted to establish LCLs from the limited amount of peripheral blood, such as in the case of starting with 0.1 ml. As a result, our findings demonstrated that the LCLs were successfully established from this small amount of blood by the simple hemolytic protocol without affecting the quality (i.e., growth and quality of DNA) of LCLs as described above. Thus, those findings should be useful for the depository facilities worldwide and researchers who are performing large-scale population studies and genetics.

From another practical point of view, there are situations in which the blood samples are not be able to be processed immediately after collection. Chang *et al*. considered and examined the blood transporting time as one of the parameters that affects the efficiency and quality of LCL establishment^16^ In this study, a total of 99 blood samples from sample groups #1 through #9 (Table 1) had a 100% success rate of LCL transformation by means of the hemolytic protocol, although these samples were collected, processed, and stored on the same day. We therefore examined the effect of keeping 5 ml or 0.1 ml of blood for 3 or 7 days at 4 °C or 25 °C prior to starting the establishment of LCLs by hemolytic protocol (Supplementary Fig. S1). As a result, we were able to establish LCLs from all of the samples. However, it took a longer period of time to transform into LCLs when the blood samples were kept for 7 days than those processed on the same day or kept for 3 days, especially when starting with a lesser volume of blood. For these reasons, we recommend to start the LCL establishment by hemolytic protocol within 3 days after receiving the blood.

In conclusion, the findings of this study demonstrated that the hemolytic protocol can serve as a reliable protocol in place of the gradient protocol for the establishment of LCLs. Of note, the hemolytic protocol enabled us to establish sufficient LCLs, even from a small amount of peripheral blood without affecting the quality of genomic DNA to be used for analysis. This simple, robust, and efficient protocol should prove to be practically useful, especially for the collection of tens of thousands of blood samples by a depository facility aimed at performing large-scale population studies.

## Methods

### Sample information

This study was approved by the Institutional Review Board of KPUM as part of ongoing studies, some of which have been published elsewhere^6^,^7^,^8^,^9^,^20^,^21^,^22^. All procedures were conducted in accordance with the tenets set forth in the Declaration of Helsinki, and written informed consent was obtained from all participating volunteers after receiving a detailed explanation of the procedures and possible consequences of being involved in the study. A total of 99 volunteers provided peripheral blood at the KPUM Hospital (Kyoto, Japan) for the present study (Table 1).

### Study design

The design of this study is shown in Fig. 1, and the sample information is shown in Table 1. Comparisons of features of prepared cells by each protocol are shown in Tables 2 and 3, and comparison of LCL growth is shown in Fig. 2. Comparisons of the quality of the genomic DNA prepared from each material using each protocol are shown in Table 4. Comparison of the concordance of genotyping results using each genomic DNA is shown in Table 5, Fig. 3, and Supplementary Table S1.

### Isolation of WBCs by the hemolytic protocol

Each volume of peripheral blood was centrifuged at 400 × g for 10 minutes at room temperature (RT). After removal of the plasma fraction, the pellet was resuspended with a five-fold amount of hypotonic erythrocyte lysis buffer (Buffer EL; Qiagen, Valencia, CA, USA) against the initial blood volume, and then incubated for 15 minutes on ice in order to burst erythrocytes. After centrifugation at 400 × g for 10 minutes at 4 °C, the supernatant containing the lysed erythrocytes was removed. The remaining pellet was then resuspended with a two-fold amount of Buffer EL against the initial blood volume, washed by centrifugation at 400 × g for 10 minutes at 4 °C. After removing the supernatant, the pellet was promptly resuspended with standard B cell culture medium consisting of Gibco™ RPMI 1640 Medium (Life Technologies, Carlsbad, CA, USA) supplemented with 10% (v/v) fetal calf serum (FCS; Life Technologies), 1 mM MEM Non-Essential Amino Acid Solution (Life Technologies), 10 mM Sodium Pyruvate (Life Technologies), 1% (v/v) Penicillin-Streptomycin (Nacalai Tesque, Inc., Kyoto, Japan), and 50 μM 2-Mercaptoethanol (Nacalai Tesque). Finally, the cell number was counted by use of a hemocytometer in order to determine the appropriate cell density for EBV infection.

### Isolation of PBMCs by the gradient protocol

Each volume of peripheral blood was diluted with an equal volume of phosphate buffered saline (PBS). The mixture was carefully layered onto an equal volume of Lymphocytes Separation Solution (Nacalai Tesque) to the initial blood volume for density gradient centrifugation. After centrifugation at 400 × g for 30 minutes at RT, the upper layer was removed and the PBMC layer was then carefully transferred into a new tube by use of a sterile pipette. The isolated PBMC fraction was then washed twice with PBS by centrifugation at 250 × g for 10 minutes at RT, and promptly resuspended with an equal volume of the standard culture medium to the initial blood volume. Finally, the cell number was then counted as described above.

### Cell count, recovery rate, viability rate, and proportion of the cell components

Suspended cells were mixed with equal volume of Turk solution (Nacalai Tesque). Next, approximately 100 cells were set into each of the four square corner areas of a glass covered Bürker-Türk hemocytometer cell counter plate, and then counted at approximately 400 cells per count. The complete blood count (CBC) of each sample was outsourced to an independent clinical laboratory (SRL, Inc., Tokyo, Japan) to be counted as described above. Based on the total number of WBC in the CBC and the isolated cells, the recovery rate (%) was calculated using the following formula:


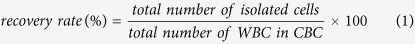


In addition, the dye exclusion test was performed using Trypan Blue Solution 0.4% (Life Technologies) stain in order to determine the number of viable cells present in the isolated cells. The viability rate (%) was calculated using the following formula:


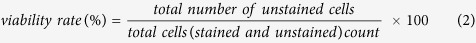


Each isolated cell was smeared on a glass slide and stained with May-Grünwald and Giemsa Dye Solution (Wako Pure Chemicals, Osaka, Japan). The stained cells were classified into the following five components: neutrophils, eosinophils, basophils, lymphocytes, and monocytes based on the standard classification under a light microscope^23^, and the percentage of each cell type included in 100 of the stained cells was then calculated.

### Preparation of EBV supernatant from the B95-8 cell line

The EBV for LCL transformation was prepared and stocked as a culture supernatant of an EBV-producing marmoset cell line, B95-8^24^, obtained from the Japan Health Sciences Foundation, Health Science Research Resources Bank (HSRRB, cell ID: JCRB9123). The maintenance of B95-8 cells and the viral supernatant were performed in accordance with the instructions from HSRRB. In brief, the B95-8 cells were cultured in Gibco™ RPMI 1640 with 10% FCS at 37 °C in 5% CO_2_. Cultured conditioned medium was recovered, all debris was depleted by centrifugation at 400 × g for 10 minutes, and the recovered supernatant was then split into aliquots and stored at −80 °C until used for infection.

### Establishment of LCL by EBV infection

LCLs were established from human peripheral blood cells or WBCs prepared by hemolytic protocol or PBMCs prepared by gradient protocol as follows. To the cells suspended in above-described standard B cell culture medium, a 50% volume of B95-8 supernatant was added to become 1.0 × 10^6^ cells/ml as the final concentration. For establishing and maintaining LCLs, 1% (v/v) phytohemagglutinin (Life Technologies) was added^25^. Cells were then cultured at 37 °C in 5% CO_2_, and one-third of the culture supernatant was replaced with fresh medium every 3-4 days. Phase-contrast microscopy revealed the transformation foci of LCLs typically at 4–7 days post infection. The success period of LCL establishment was defined as the day when the diameter of transformed cell clusters reached 50 μm. In order to evaluate the cell growth, the number of cells was counted at 2-week intervals until 8 weeks.

### SNP genotyping and assessment of genotype concordance

Twenty-four samples were used from sample groups #7, #8, and #9 (Table 1c). Genomic DNA was extracted using the genomic BioRobot^®^ EZ1^™^ Robotic Liquid Handler (Qiagen) in accordance with the manufacturer’s instructions. The yield of DNA extraction and the ratio of A260/280 absorbance of genomic DNA was then measured and calculated (Table 4). The GeneChip^®^ Mapping 100 K Array Set was used in accordance with the manufacturer’s instructions, and genome-wide genotype data of 116,204 SNPs was then obtained for each sample. The SNP genotype data derived from each group was then compared and summarized as a cross-classification table (Table 5). The concordance rate of 348,612 (116,204 SNPs × 3 samples) SNP genotype calls was calculated using the following formula:


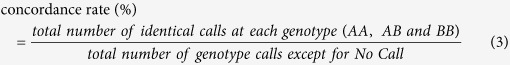


Kappa statistics was applied to objectively assess the concordance rate. In addition, the pairwise distances between the SNP genotype data obtained from genomic DNA derived from peripheral blood and that obtained from genomic DNA derived from LCL-hemolytic was examined using PLINK open-source whole genome association analysis toolset software with the “–cluster” option (http://pngu.mgh.harvard.edu/~purcell/plink/), which is commonly used in genetics for calculating identity-by-state distances (Fig. 3). The value of pairwise distance indicates 0 if the pair is an identical twin (i.e., expected that all the genotypes to be fully matched). The number of SNPs to calculate pairwise distance was compared by the condition of SNP filtering based on the call rate as: no filter (i.e., all SNPs were applied), 95%, and 99% (i.e., the number of genotype with no call was less than 5% or 1% in each SNP, respectively; the regular filtering condition applied in GWAS). Additional information of the DNA microarray experiments and comparison processes are described in the Supplementary Note.

### Statistical analysis

R software (http://www.r-project.org/) was used for all statistical analyses (i.e., Shapiro-Wilk test, Wilcoxon signed rank test, Wilcoxon rank sum test, and Kappa statistics) and for creating graphs. In addition, the R package of “exactRankTests” was used for the Wilcoxon tests. To manage the genotype data, our in-house TG Server System based on the Labo Server Software System (World Fusion, Tokyo, Japan) was used as previously described^6^,^7^,^8^,^9^. Genotype concordance estimated by pairwise distance was analyzed by use of PLINK.

## Additional Information

**How to cite this article:** Omi, N. *et al*. Efficient and reliable establishment of lymphoblastoid cell lines by Epstein-Barr virus transformation from a limited amount of peripheral blood. *Sci. Rep.* **7**, 43833; doi: 10.1038/srep43833 (2017).

**Publisher's note:** Springer Nature remains neutral with regard to jurisdictional claims in published maps and institutional affiliations.

## Supplementary Material

Supplementary Information

## Figures and Tables

**Table 1:** Sample information.

Comparative experiment	Sample group	Blood volume (ml)	Participated volunteers (n)	Mean age at sampling (age range)	Sample number (n)		
					Peripheral blood	Hemolytic	Gradient
(a)	#1	5	20	64.3 (34–82) years	0	20	20
	#2	2	20	64.6 (30–86) years	0	20	20
	#3	0.1	20	69.7 (46–91) years	0	20	20
(b)	#4	5	10	61.7 (51–70) years	0	10	10
	#5	2	10	67.8 (57–76) years	0	10	10
	#6	0.1	10	67.3 (55–86) years	0	10	10
(c)	#7	5	3	52.0 (28–66) years	6*	3	3
	#8	2	3	72.0 (69–76) years	3	3	0
	#9	0.1	3	67.7 (65–69) years	3	3	0

^*^(3 blood samples from sample group #7) × (each technical replicate) = 6 microarray samples.

**Table 2:** Comparison of recovery and viability of isolated cells between the two protocols.

Sample group	Blood volume (ml)	Total WBC count in CBC (10^3^/μl)^*^	Isolated WBCs by the hemolytic protocol		Isolated PBMCs by the gradient protocol		Viability comparison **P**-value^***^
			Recovery^**^	Viability^**^	Recovery^**^	Viability^**^	
#1	5	6.2 ± 1.2	47.4 ± 3.8	97.7 ± 1.3	18.4 ± 1.8	98.4 ± 1.7	0.1140
#2	2	5.6 ± 1.4	46.1 ± 4.1	97.1 ± 1.2	16.2 ± 2.1	98.1 ± 3.5	0.1925
#3	0.1	5.7 ± 1.3	41.6 ± 4.4	96.6 ± 1.5	14.6 ± 2.4	98.8 ± 5.6	0.0012

^*^Values indicate the mean ± SD of total cell number of WBC in the CBC.

^*^Values represented as the mean ± SD of the percentage.

^***^P-values indicate the comparison between the hemolytic and the gradient protocol by Wilcoxon signed-rank test.

**Table 3:** Proportion of cell components of the isolated cells.

Protocol	Sample group	Blood volume (ml)	WBCs				
			Granulocytes*			PBMCs*	
			Neutrophils	Eosinophils	Basophils	Lymphocytes	Monocytes
Hemolytic protocol	#1	5	52.8 ± 5.7	2.1 ± 1.3	0.4 ± 0.5	43.6 ± 5.3	1.2 ± 0.7
	#2	2	53.8 ± 6.9	1.5 ± 1.2	0.6 ± 0.5	43.1 ± 6.5	1.1 ± 0.8
	#3	0.1	52.3 ± 4.5	3.4 ± 1.7	0.6 ± 0.5	42.8 ± 4.7	1.1 ± 0.8
Gradient protocol	#1	5	8.2 ± 3.5	0.8 ± 0.7	0.2 ± 0.4	90.2 ± 4.3	0.9 ± 0.6
	#2	2	15.6 ± 5.0	0.8 ± 0.6	0.2 ± 0.4	82.6 ± 5.5	0.9 ± 0.7
	#3	0.1	30.2 ± 6.9	1.6 ± 0.8	0.3 ± 0.5	67.2 ± 7.1	0.8 ± 0.6

^*^Values represent the mean ± SD of the percentage of the cells in each sample group.

**Table 4:** Quality and quantity of genomic DNA for genotype concordance analyses.

Sample group	Blood volume (ml)	DNA source	−80 °C period (weeks)	Cell number when thawed (×10^6^)	Viability when thawed (%)	Culture period (weeks)****	Final cell number (×10^6^)	DNA yield (μg)	OD260/280
#7	5	Peripheral blood^*^	136**	—	—	—	—	4.2 ± 1.9	1.79 ± 0.09
		LCL-gradient	130***	1.2 ± 4.9	79.2 ± 4.7	6	2.3 ± 0.4	3.2 ± 0.7	1.96 ± 0.02
		LCL-hemolytic	130***	1.1 ± 2.2	80.2 ± 5.5	6	3.0 ± 0.4	3.8 ± 1.3	1.89 ± 0.01
#8	2	Peripheral blood^*^	60**	—	—	—	—	3.2 ± 2.2	1.82 ± 0.03
		LCL-hemolytic	52***	0.4 ± 0.4	78.9 ± 8.3	8	2.0 ± 1.0	2.6 ± 1.2	1.93 ± 0.04
#9	0.1	Peripheral blood^*^	10**	—	—	—	—	3.7 ± 3.5	1.82 ± 0.04
		LCL-hemolytic	0	—	—	12	0.7 ± 0.2	1.7 ± 0.6	1.88 ± 0.01

^*^350 ul of peripheral blood was used as a source of DNA.

^**^Genomic DNA was stored at −80 °C.

^***^LCLs were stored at −80 °C after 2 weeks culture from EBV infection.

^****^Total cultivation time including 2 weeks culture before cryopreservation.

Except for the “−80 °C period” and the “Cultivation period”, the values represent the mean ± SD.

**Table 5:** Cross-classification of genotype calls for evaluating the protocols started from 5 ml of peripheral blood.

Compared sample		Peripheral blood from sample group #7 (All SNPs)					Concordance (%)	Kappa**
		AA	AB	BB	No call	Total		
Peripheral blood from sample group #7 (as technical replicate)	AA	127,257	57	0	626	127,940		
	AB	129	89,987	88	545	90,749		
	BB	0	61	126,041	622	126,724		
	No Call	936	509	859	895	3,199		
	Total	128,322	90,614	126,988	2,688	348,612*	99.90	0.9985
LCL-hemolytic from sample group #7	AA	127,613	68	0	647	128,328		
	AB	80	90,031	62	506	90,679		
	BB	0	78	126,305	644	127,027		
	No Call	629	437	621	891	2,578		
	Total	128,322	90,614	126,988	2,688	348,612*	99.92	0.9987
LCL-gradient from sample group #7	AA	127,139	52	0	568	127,759		
	AB	125	89,964	140	602	90,831		
	BB	0	77	125,774	548	126,399		
	No Call	1,058	521	1,074	970	3,623		
	Total	128,322	90,614	126,988	2,688	348,612*	99.89	0.9983

^*^Affy100k Array (116,204 SNPs) × 3 samples = 348,612 SNPs.

^**^Closer to 1.0 indicates higher reproducibility.
