# Supplementary material for: Efficient and reliable establishment of lymphoblastoid cell lines by Epstein-Barr virus transformation from a limited amount of peripheral blood
Source: Sci Rep. 2017 Mar 8;7:43833. doi: 10.1038/srep43833 (PMC5341036; doi:10.1038/srep43833)
Supplement: Supplementary Information [file srep43833-s1.pdf]

Supplementary Information

Efficient and reliable establishment of lymphoblastoid cell lines by Epstein-Barr virus transformation from a limited amount of peripheral blood

Natsue Omi[1], Yuichi Tokuda[1], Yoko Ikeda[2], Mori Ueno[2], Kazuhiko Mori[2], Chie Sotozono[2], Shigeru Kinoshita[2,3], Masakazu Nakano[1], & Kei Tashiro[1]

[1]Department of Genomic Medical Sciences, Kyoto Prefectural University of Medicine, Kyoto, Japan.
[2]Department of Ophthalmology, Kyoto Prefectural University of Medicine, Kyoto, Japan.
[3]Department of Frontier Medical Science and Technology for Ophthalmology, Kyoto Prefectural University of Medicine, Kyoto, Japan.

Corresponding author, E-mail: tashiro@koto.kpu-m.ac.jp

Contents

Page 2: Supplementary Table S1.

Page 3: Supplementary Figure S1.

Page 4: Supplementary Figure S2.

Page 5: Supplementary Figure S3.

Pages 6 and 7: Supplementary Note.

Supplementary Table S1. Cross-classification of genotype calls for evaluating the starting peripheral blood volume in case of hemolytic protocol

Sample group	Compared sample		Peripheral blood genotype calls (All SNPs)					Conc. (%)	Kappa†
			AA	AB	BB	NoCall	Total		
#8	LCL-Hemolytic from 2 ml	AA	128,680	111	0	893	129,684		
		AB	50	88,140	55	414	88,659		
		BB	1	114	126,737	1018	127,870		
		NoCall	492	591	453	863	2,399		
		Total	129,223	88,956	127,245	3,188	348,612*	**99.90**	**0.9985**
#9	LCL-Hemolytic from 0.1 ml	AA	126,658	189	0	1638	128,485		
		AB	95	88,794	109	551	89,549		
		BB	0	228	125,431	1521	127,180		
		NoCall	764	731	870	1033	3,398		
		Total	127,517	89,942	126,410	4,743	348,612*	**99.82**	**0.9972**

*Affy100k Array (116,204 SNPs) × 3 samples = 348,612 SNPs

†Closer to 1.0 indicates higher reproducibility.

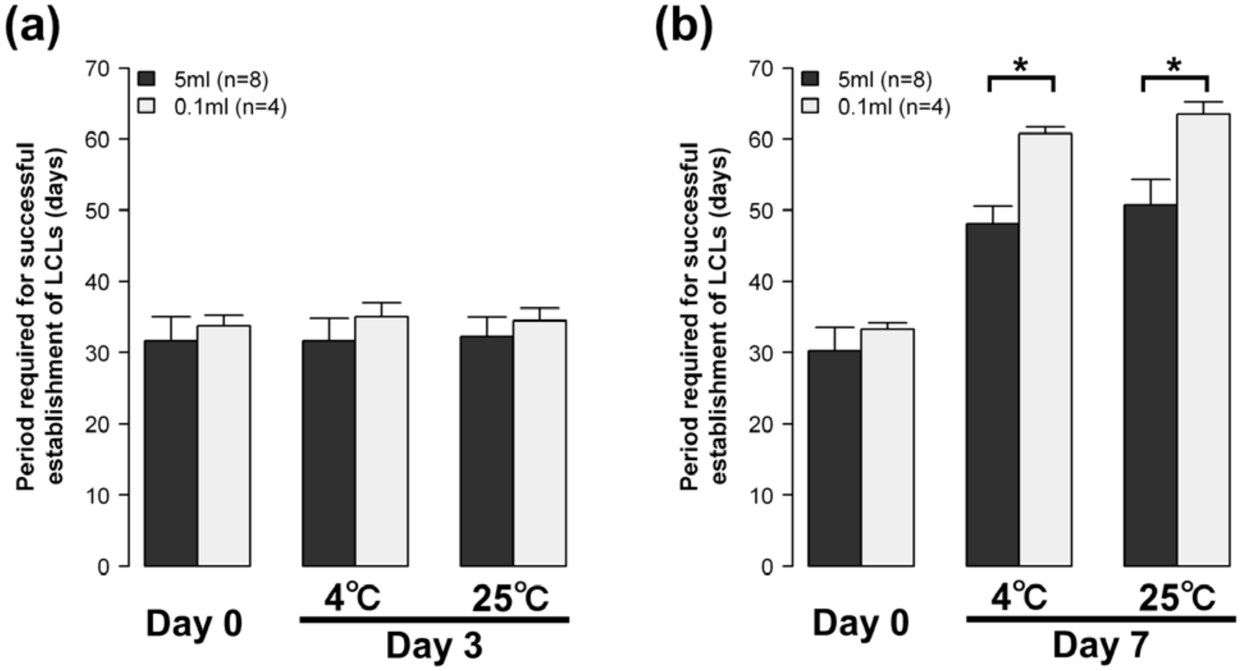

Supplementary Figure S1. Effect of keeping blood in different conditions before establishing LCLs.

The bar plots show the mean and SD of the days required for the successful establishment of LCL from the day when the blood was processed by the hemolytic protocol. The black and gray colored bars indicate the different volume of starting blood, i.e., 5 ml and 0.1 ml, respectively. Day 0 is the day that the blood sample was obtained from the volunteer subjects, processed by the hemolytic protocol, and infected with EBV, which is the routine procedure in our laboratory. All samples used in this analysis were independent from the samples used in the other analysis of this manuscript. Asterisks denote the *P*-value of the statistical significance ($P<0.01$) by the Wilcoxon rank sum test. (**a**) Effect of keeping 3-days in different conditions. Day 0 samples were compared with the same blood samples stored for 3 days (Day 3) before processing. The mean ages (age range) of the volunteers in the 5 ml and 0.1 ml peripheral blood sample groups were 65.1 (47-87) and 69.5 (66-74), respectively. (**b**) Effect of keeping 7-days in different conditions. The mean ages (age range) of the volunteers in the 5 ml and 0.1 ml peripheral blood sample groups were 70.0 (65-75) and 72.0 (66-79), respectively.

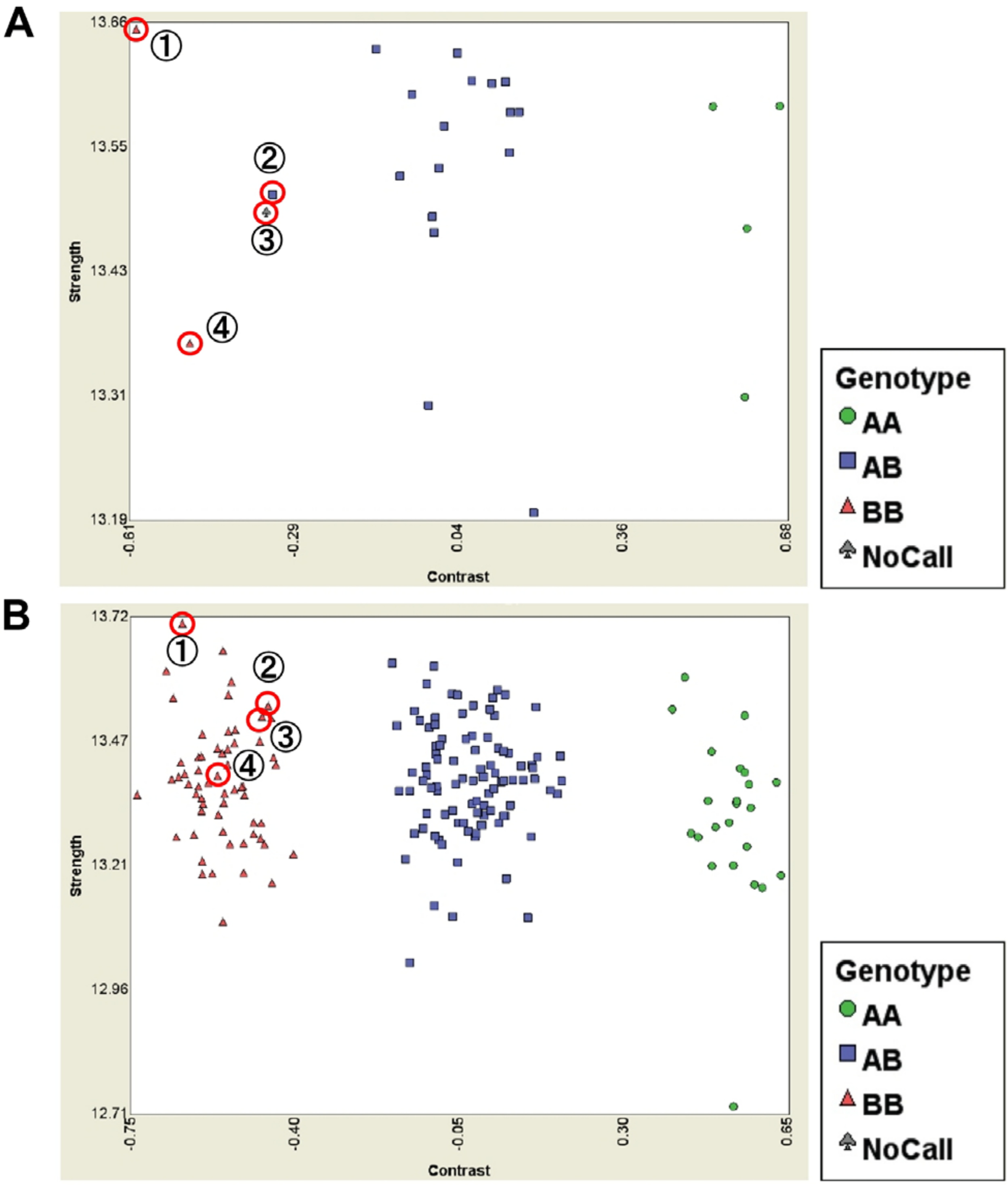

Supplementary Figure S2. A clustering result of a SNP genotype call.

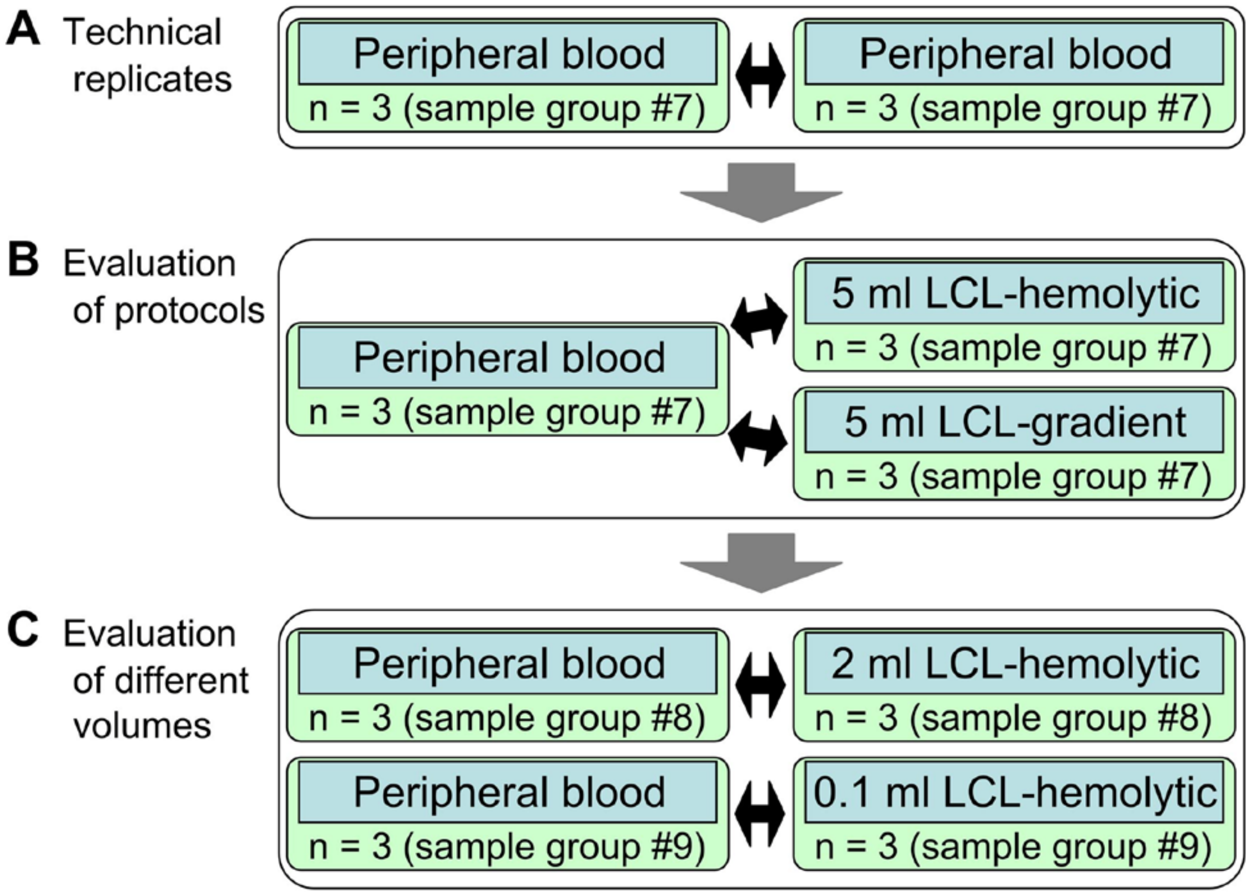

Supplementary Figure S3. Three steps for genotype data comparison.

Supplementary Note

Analysis of LCL genome stability.

1. Genotype data preparation

As described in the main manuscript, genomic DNA from peripheral blood was extracted by using the genomic BioRobot® EZ1™ Robotic Liquid Handler (Qiagen, Valencia, CA, USA). In addition, the genomic DNA from each established LCL (LCL-hemolytic or LCL-gradient) was extracted via the same method (Table 4). Twenty-four DNA samples from sample groups #7, #8, and #9 were applied to the following experiments (Table 1c). Each genomic DNA was adjusted to 50 ng/μl based on the DNA concentration measured by NanoDrop ND-1000 Spectrophotometer (Thermo Fisher Scientific, Inc., Wilmington, DE, USA). The genome-wide genotype data of SNPs was obtained by using DNA microarray, GeneChip® Mapping 100K Array Set, consisted of Xba-array and Hind-array (Affy100k; Affymetrix, Inc., Santa Clara, CA, USA) according to the manufacturer's instructions. Briefly, 250 ng of each genomic DNA was applied to both the Xba- (58,960 probes) and Hind- (57,244 probes) arrays for hybridization; therefore, genotype data by Affy100k was obtained for a total 116,204 SNPs per one sample.

The raw data set of the hybridization results was calculated by genotype caller program, which integrates the intensity signal data of each sample and makes the cluster to divide into three types of genotype (i.e., AA, AT, and TT) or judges as "No Call" when the clustering failed. In order to improve the accuracy of genotype calling based on the clusters, we added the genotype data set of Affy100k, which was derived from 155 DNA samples from our other study (unpublished data) and passed the quality control (≥ 95% of the genotype call rate per sample), to the program. These additional genotype data improved the cluster for genotype calling as shown in Supplementary Figure S2, which shows the typical example of clustering result for a SNP with or without increasing the number of genotype data. If only 24 samples were clustered, the genotype result of a sample marked as "2" had been separately clustered from that of "1" and "4". If only 24 samples were clustered, the genotype result of the other sample marked as "3" had been "No Call" (Supplementary Fig. S2A). However, after adding 155 samples, all of these genotypes were fit into a single cluster as the same genotype (Supplementary Fig. S2B). Consequently, the data set derived from a total of 179 samples was used to generate the original genotype cluster for our population. Each genotype of SNP was called by using the BRLMM algorithm build in Genotyping Console™ software (Affymetrix).

2. Evaluation process

In order to evaluate the quality of genomic DNA from LCLs, which are infected and transformed by EBV, concordance of 348,612 SNP genotype data between established LCLs and the peripheral blood was assessed as shown in Supplementary Fig. S3.

We first obtained SNP genotype data derived twice from the same samples in order to evaluate the genotype data concordance of technical replicates and determine the error rate that occurred in the normal experimental processes (Supplementary Fig. S3A). This step was performed by using 3 pairs of the data set derived from three samples in sample group #7 (Table 1c).

Next, the SNP genotype data derived either from LCL-hemolytic or LCL-gradient was compared with the SNP genotype data derived from the peripheral blood, from which LCLs were established (Supplementary Fig. S3B). This step was performed by using 3 sample pairs from sample group #7 (Table 1c), i.e., 9 samples (i.e., each 3 of parental blood, LCL-hemolytic, and LCL-gradient) in total, were applied to the comparison. In these experiments, 5 ml of peripheral blood was used as the starting material.

Finally, to investigate the influence of the starting volume of blood for LCL establishment, the SNP genotype data obtained from LCL-hemolytic started from 2 ml or 0.1 ml of peripheral blood was compared with the SNP genotype data derived from the peripheral blood, from which LCLs were established (Supplementary Fig. S3C). Each of 3 samples started with 2 ml or 0.1 ml of peripheral blood were applied to this evaluation from the sample groups #8 and #9, respectively (Table 1c).

3. Evaluation results

The concordance rate of technical replicates (Supplementary Fig. S3A) was 99.90% (Table 5), indicating that the error rate of about 0.1% could spontaneously occur during the 100K microarray genotype experiments.

Given the above background technical replication data, the concordance rates between the data from genomic DNA from LCLs and the data from peripheral blood DNA (Suppl. Fig. S3B) were extremely high (approximately 99.90%) in both the hemolytic and gradient protocols (Table 5). Moreover, when using the hemolytic protocol, the concordance rate remained constant when the starting volume was reduced to 2 ml (99.90%), and remained high enough even when reduced down to 0.1 ml (99.82%) (Supplementary Fig. S3C, Supplementary Table S1). These results were also supported by the Kappa statistics (Table 5, Supplementary Table S1).

Taken together, these results suggest that the genomic DNA derived from LCLs established by the hemolytic protocol had a minimum effect of EBV transformation and was sustainable for practical use.
